# Management of Community-Acquired Pneumonia in Pediatrics: Adherence to Clinical Guidelines

**DOI:** 10.3389/fped.2020.00302

**Published:** 2020-06-19

**Authors:** Rim Tannous, Raymond N. Haddad, Paul-Henri Torbey

**Affiliations:** ^1^Faculty of Medicine, Saint-Joseph University, Beirut, Lebanon; ^2^Department of Pediatrics, Hotel Dieu de France University Medical Center, Saint Joseph University, Beirut, Lebanon; ^3^Division of Pediatric Pulmonology, Department of Pediatrics, Hotel Dieu de France University Medical Center, Saint Joseph University, Beirut, Lebanon

**Keywords:** pneumonia, pediatrics, guidelines & recommendations, clinical practices assessment, public health

## Abstract

**Objectives:** To evaluate adherence to guidelines for inpatient care of pediatric patients with community-acquired pneumonia (CAP).

**Background:** Pediatric CAP is one of the most common acute infections requiring hospital admission. Discrepancies between recommended care and effective management are reported, raising the necessity to evaluate our local clinical practices.

**Patients and Methods:** Retrospective data review of all children hospitalized for CAP at our institution was conducted between 2014 and 2017. Adherence to inpatient care guidelines was evaluated with a focus on indication of hospitalization, initial antibiotic choice, treatment duration, and hospital stay. A bivariate analysis was performed to identify clinical factors influencing adherence rates.

**Results:** A total of 122 children (median age of 3.5 years) were identified. Hospital admission was indicated in 47.5% of patients and was driven by the value of serum CRP as well as prolonged fever. Median hospital stay was 4 days and was justified in 23.8% of patients. The choice of antibiotics was relevant in 91.8% of cases and amoxicillin-clavulanate was the most prescribed drug. The drugs dose, interval, and route of administration were respected in all cases. Antimicrobial therapy lasted for a median of 10 days and was in accordance with recommendations in 58.3% of patients. No clinical parameter was found to be significantly associated with length of stay or choice and duration of treatment.

**Conclusions:** The choice of antibiotics was consistent with guidelines but treatment duration, indication and length of hospitalization still need to be improved.

## Introduction

Community-acquired pneumonia (CAP) is a highly prevalent infection and remains the first cause of early childhood mortality in developing countries (1–3). Scientific guidelines were established to optimize and standardize medical care yet, several authors reported discrepancies between recommendations and actual management (4–8), leading to overuse of antibiotics, a higher resistance rate in community-acquired pathogens (9), and longer hospitalizations (7). This study aims to evaluate our local inpatient practices in children with CAP and to identify clinical parameters likely to influence adherence to guidelines, in order to better understand eventual gaps in our medical approaches.

## Patients and Methods

### Study Population and Design

Between January 2014 and January 2017, all patients aged from 1 month to 15 years, and hospitalized for CAP at the Saint Joseph university teaching Hospital, Hotel Dieu de France were retrospectively reviewed and included in this study. The diagnosis of CAP was strictly based on radiological findings confirmed by the attending radiologist and pediatric pulmonologist. Complicated pneumonia was defined by the presence of pleural effusion and/or pulmonary abscess. All relevant clinical parameters and laboratory findings were collected. Personal history and underlying diseases were also recorded. The severity of the disease was assessed according to the British guidelines (10, 11). Adherence to the institution's protocol regarding inpatient CAP management was evaluated with a focus on the indication of hospitalization, initial antibiotic choice, length of treatment, and hospital stay duration. Our protocol was adopted from the Pediatric Infectious Diseases Society and Infectious Diseases Society of American guidelines (12, 13) with the only difference being that amoxicillin-clavulanate (AC) was advised as the narrow-spectrum first-line therapy.

### Statistical Analysis

Statistical analyses were performed using the Statistical Package for the Social Sciences Statistics (SPSS), version 22 for Macintosh (IBM, Armonk, NY, United States). Categorical variables were reported as frequency and percentage. Continuous variables as mean with standard deviation or median with range, depending on normality of distribution. The normality of measurements was assessed using skewness and kurtosis values and supported by the Shapiro–Wilk test. Statistical analysis of the categorical variables were conducted using Chi-square test and Fisher's exact test as appropriate and by *t*-test and Mann–Whitney U test for continuous variables. A *p*-value < 0.05 was considered statistically significant. All reported *P*-values are two-sided.

## Results

**Chart 1:**
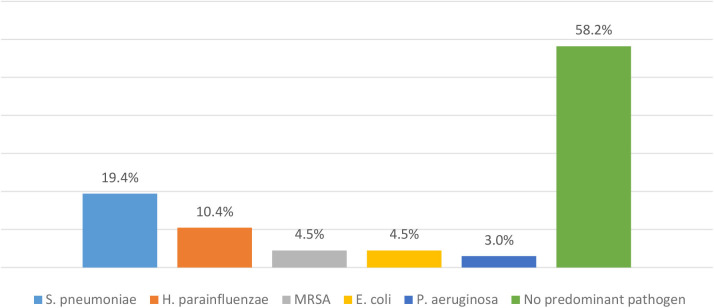
Pathogens detected in sputum culture of children hospitalized for CAP, *n* = 67. MRSA, Methicillin-Resistant *Staphylococcus Aureus*; *S. pneumoniae, Streptococcus pneumoniae*; *H. parainfluenzae, Haemophilus parainfluenzae*; *E. coli, Escherichia coli*; *P. aeruginosa, Pseudomonas aeruginosa*.

**Chart 2:**
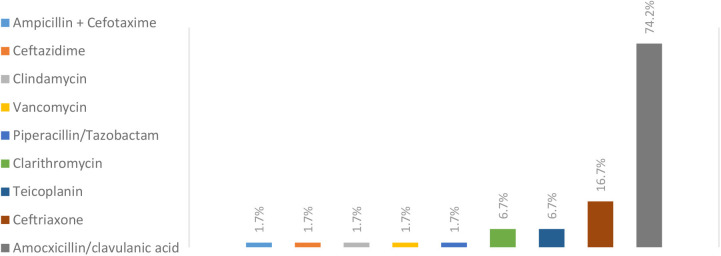
Prescribed antibiotics for inpatient treatment of pediatric CAP, *n* = 120.

A total of 122 children (53.3% boys) were included. Patients' median age was 3.5 years. Patients' characteristics are summarized in Table 1. Among children hospitalized for CAP, 31% had underlying diseases: asthma (14.8%), immunodeficiency (5.7%), gastro-esophageal reflux disease (5.7%), congenital heart defect (4.9%), neuromuscular disease (4.1%) and broncho-pulmonary dysplasia (2.5%). Pneumonia was classified severe in 39.3% and complicated in 14.8% of cases. Four children required intensive care according to guidelines with no reported death. Hospitalization lasted for a median of 4 days and the median duration of treatment was 10 days. Testing for viral pathogens (influenza and respiratory syncytial virus) was carried out in 36.9% of cases and most of them came negative. Bacterial sputum cultures were done in 55% of cases and results are summarized in Chart 1. Blood cultures were performed in 28.7% of cases and almost all were negative. Different antibiotics were used but the most prescribed one was AC (Chart 2). The choice of antibiotic therapy was relevant in 91.8% of cases. The drugs' dose, interval, and route of administration were respected in all cases. Hospital admission was indicated in only 47.5% of patients and was found to be driven by the value of serum C-reactive protein (CRP) and prolonged fever. No clinical factor was shown to significantly affect hospital stay duration nor the choice and duration of antibiotic therapy as shown in Tables 2, 3.

**Table 1:** Patients characteristics.

	***N* = 122**
Male, *N (%)*	65 (53.3)
Age (years)*, Median (range)*	3.5 (0.1–14)
≤ 1 year, *N (%)*	20 (16.4)
>1 year, *N (%)*	102 (83.6)
Recurrent pneumonia, *N (%)*	28 (23.0)
Positive neonatal history^1^, *N (%)*	11 (9.0)
Underlying diseases^*^, *N (%)*	38 (31.1)
Asthma	18 (14.8)
Immunosuppression	7 (5.7)
Gastroesophageal reflux disease	7 (5.7)
Congenital heart defect	6 (4.9)
Neuromuscular disease	5 (4.1)
Broncho-pulmonary dysplasia	3 (2.5)
Severity of pneumonia, *N (%)*	
Mild to moderate	74 (60.7)
Severe	48 (39.3)
Complicated pneumonia*, N (%)*	18 (14.8)
Symptoms at presentation	
Signs of respiratory distress, *N (%)*	29 (23.8)
Cyanosis, *N (%)*	4 (3.3)
Trans-capillary saturation (%)*, Median (range)*	97 (80–100)
Altered state of consciousness, *N (%)*	19 (15.6)
Temperature at home (°C)*, M ± SD (range)*	39 ± 0.8 (37–40.6)
Fever, *N (%)*	109 (89.3)
High grade fever (>39°C), *N (%), n* = 109	46 (42.2)
Fever duration before admission (days)*, Median (range)*	3 (1–20)
<4 days*, N (%)*	66 (54.1)
White blood cells*, M ± SD (range)*	13,000 ± 6,500 (2,800–35,500)
CRP*, Median (range)*	33 (3–439)
Procalcitonin*, Median (range)*, n=35	0.3 (0–225)
Chest X-ray findings, *N (%)*	
Multilobar infiltrates	23 (23.8)
Pleural effusion	18 (14.8)
Antibiotics, *N (%)*	120 (98.4)
Narrow spectrum	92 (76.7)
Broad-spectrum	27 (22.5)
Upgrade in therapy	20 (16.7)
Pediatric intensive care unit, *N (%)*	4 (3.3)
Endotracheal intubation, *N (%)*	2 (1.6)
Fever duration from initiation of treatment to defervescence (days)*, Median (range)*	1 (1–31)
<4 days*, N (%)*	109 (89.3)
Hospital stay duration(days)*, Median (range)*	4 (2–41)
Duration of antibiotic therapy (days)*, Median (range), n* = 120	10 (7–50)
≤ 10 days*, N (%)*	64 (53.3)
≤ 14 days*, N (%)*	107 (89.2)

1 *Prematurity and/or neonatal respiratory distress syndrome*.

M ± SD: mean ± standard deviation.

* *More than one choice applied*.

**Table 2:** Distribution of adherence to recommendations' rates in children hospitalized for CAP according to clinical factors.

	**Adherence to recommendations**			
	**Indication for hospitalization, *n* = 122**	**Choice of antibiotics, *n* = 120**	**Hospital stay duration, *n* = 122**	**Antibiotic therapy duration, *n* = 120**
Total	58 (47.5)	110 (91.8)	29 (23.8)	70 (58.3)
**Age groups**				
≤ 1 year	16 (80.0)	17 (85.0)	7 (35.0)	14 (70.0)
>1 year	42 (41.2)	93 (93.0)	22 (21.6)	56 (56.0)
*p*-value	**0.003^a^**	0.368^b^	0.250^b^	0.323^a^
**Neonatal history^1^**				
Positive	8 (72.7)	10 (100.0)	3 (27.3)	6 (60.0)
Negative	50 (45.0)	100 (90.9)	26 (23.4)	64 (58.2)
*p*-value	0.114^a^	1.000^b^	0.722^b^	1.000^b^
**Recurrent pneumonia**				
Yes	15 (53.6)	23 (85.2)	9 (32.1)	12 (44.4)
No	43 (45.7)	87 (93.5)	20 (21.3)	58 (62.4)
*p*-value	0.522^a^	0.230^b^	0.311^a^	0.122^a^
**Pneumonia severity**				
Mild to moderate	10 (13.5)	64 (86.5)	13 (17.6)	43 (58.1)
Severe	48 (100.0)	46 (100.0)	16 (33.3)	27 (58.7)
*p*-value	**<0.0001**^**a**^	**0.013**^**b**^	0.053^a^	1.000^a^
**Complicated pneumonia**				
Yes	10 (55.6)	6 (85.7)	4 (22.2)	10 (58.8)
No	48 (46.2)	11 (55.0)	25 (24.0)	60 (58.3)
*p*-value	0.610^a^	0.204^b^	1.000^b^	1.000^a^
**Underlying diseases**				
Yes	25 (65.8)	35 (92.1)	9 (23.7)	22 (57.9)
No	33 (39.3)	75 (91.5)	20 (23.8)	48 (58.5)
*p*-value	**0.01**^**a**^	1.000^b^	1.000^a^	1.000^a^

1 *Prematurity and/or neonatal respiratory distress syndrome*.

a *Chi-square test*;

b *:Fisher test*.

*Bold values are significant p-values*.

**Table 3:** Clinical factors affecting adherence to recommendations in children hospitalized for CAP.

	**WBC count**	**Serum CRP**	**Fever duration before admission (days)**	**Fever duration from initiation of treatment to defervescence (days)**
	**M** **±** **SD**	**Median (range)**		
**Criteria for hospitalization**				
Respected	11,800 ± 5,700	25 (3–439)	2 (1–20)	**NP**
Not respected	14,000 ± 7,000	52 (3–407)	4 (1–10)	
*p*-value	0.054^a^	**0.007^b^**	**0.007^b^**	
**Choice of Antibiotics**				
Respected	13,100 ± 6,800	35 (3–439)	3 (1–20)	**NP**
Not respected	12,100 ± 4,300	38 (3–301)	3 (1–7)	
*p*-value	0.645^a^	0.621^b^	0.768^b^	
**Hospital stay**				
Respected	11,200 ± 6,300	48 (3–439)	4 (1–7)	**NP**
Not respected	13,500 ± 6,500	31 (3–407)	3 (1–20)	
*p*-value	0.080^a^	0.645^b^	0.410^b^	
**Antibiotic therapy duration**				
Respected	13,000 ± 6,500	29 (3–439)	3 (1–20)	1 (1–31)
Not respected	12,500 ± 6,700	50 (3–407)	4 (1–10)	1 (1–7)
*p*-value	0.489^a^	0.114^b^	0.166^b^	0.928^b^

*M ± SD, mean ± standard deviation; NP, not performed; WBC, white blood cell; CRP, C-reactive protein*.

a *T-test*;

b *Mann-Whitney U test*.

*Bold values are significant p-values*.

## Discussion

### Antimicrobial Therapy: Choice and Duration

Since the publication of PIDS/IDSA guidelines in 2011 (12), many authors examined adherence of pediatricians to these guidelines that were set in the first place to warrant better clinical outcomes, and reduce management discrepancies. In our study, 74.2 % of patients with bacterial CAP were treated with AC while broad-spectrum agents such as vancomycin, clindamycin, third-generation cephalosporin were used in the remaining cases. *Streptococcus pneumoniae* was found to be the most prominent pathogen as reported in other studies (14, 15). The local epidemiology of invasive pneumococcal strains documents penicillin resistance, prevalence ranging from 40 to 50% (16–18) with similar pneumococcal susceptibility profiles in regional countries (19–21). Moreover, although Queen et al. demonstrated that narrow-spectrum antibiotic therapy was not inferior to broad-spectrum in all measured outcomes including length of stay and duration of fever (15), guidelines remain not always respected (7, 21–23). Ambroggio et al. reported the use of broad-spectrum therapy in up to 93% of hospitalized CAP across 33 pediatric hospitals (22) while Hersh et al. reported that considerable variation in empiric prescribing patterns is found even among pediatric infectious disease physicians (23). Recommendations concerning antibiotic choice were respected in 91.8% of our cases, and neither white blood cell count nor serum CRP values affected the choice of antibiotics. This represents a satisfactory finding when compared to the studies cited above. However, our analysis showed a significantly lower adherence rate to recommendations of antibiotic choice when dealing with mild to moderate pneumonia in comparison to severe cases of pneumonia, pointing to the necessity of additional improvements.

Our results showed that antimicrobial therapy lasted for a median of 10 days and was in 58.3% of cases, in accordance with our protocol that recommends a 7–10 days course. According to the American guidelines, there is no recommended strict treatment length but a 10 days course is considered as acceptable and could be prolonged when dealing with certain pathogens or when clinical or radiological deterioration occurs during hospital stay (12, 13). World Health Organization (WHO) guidelines recommend a shorter course for patients aged <5 years (24). Several investigations on the treatment of pediatric pneumonia showed no significant difference between short and long courses neither in clinical cure rates nor in treatment failure or relapse rates (25, 26). With this in mind, we did not identify clinical parameters significantly associated with the treatment length, leaving us with a strong belief that longer courses were prescribed to warrant the minimal required duration for non-compliant families that usually tend to stop treatment after hospital discharge and upon clinical improvement.

### Hospitalization: Indication and Duration

Hospitalization was indicated in only 47.5% of cases with expected higher rates of non-adherence to recommendations in older children, patients with non-severe clinical presentations and those with no underlying diseases; a category of patients suitable for outpatient care. Indication of hospitalization was also not respected when higher values of CRP and longer febrile illnesses were recorded. Higher CRP values are more likely to be associated with bacterial pneumonia with a variable cut-off between different studies (27–29). According to a meta-analysis published in 2008, children with bacterial pneumonia had a significantly higher probability of having CRP values of 35–60 mg/L on admission, compared to children with viral pneumonia (29). Although these findings do not solely explain unjustified hospital admissions, we believe that attending pediatricians are likely to be influenced by the preferences of the family. Parental understanding of CRP is recently increasing and caregivers tend to consider higher CRP values as a sign of severity and a strong marker of infection, especially in recurrent illnesses. Caregivers are often concerned about being far from academic hospitals and their inability to provide adequate home surveillance leading to excessive hospitalizations (5). Therefore, a useful initiative would be to raise awareness among families on nosocomial risks of inpatient treatment.

Hospital stay lasted for a median of 4 days, slightly longer than the duration reported by Jain et al. (14). It turned out that it does not always comply with established recommendations and can be affected, among other causes, by time-consuming administrative procedures that physicians tend to overlook (30, 31). Unjustifiable days being waiting for diagnostic or therapeutic measures stand out as logical reasons behind patients' discharge delay especially when our analysis did not identify any associated clinical or biological factors. Efforts should be toward strategies likely to improve the internal processes of the hospital, which mostly could be prevented through appropriate management, thus decreasing inappropriate hospital stay and preserving hospital resources for patients who need them.

### Study Limitations and Strengths

This is a retrospective study with a relatively limited number of participants and a wide age range. Data were collected from a single-center, which makes it difficult to extrapolate results to other populations, in addition to differences in implanted guidelines among hospitals. The strength of this study resides in the detailed data collection, precise radiological and laboratory diagnosis and the in-depth review of this relevant topic, rarely discussed in our country and in the Arab world. Our results also highlight the characteristics of Lebanese children admitted for CAP, as our population comes from all over the country.

## Conclusion

Despite well-established guidelines for the management of pediatric CAP, lack of adherence regarding the indication of inpatient care, length of stay and treatment duration remains frequently observed even in an academic hospital. Clinical practice assessments is the major key to identify potential rationales, to sensibilise healthcare professionals to major issues and to secondarily implement effective measures likely to improve the quality of medical care.

## Data Availability Statement

The raw data supporting the conclusions of this article will be made available by the authors, without undue reservation, to any qualified researcher.

## Ethics Statement

The authors assert that all procedures contributing to this work comply with the Helsinki Declaration of 1975, as revised in 2008, and has been approved by the Saint Joseph University Research Ethics Committee on Human Clinical Research (CEHDF 1523). Written informed consent was signed by the patients' legal guardians to analyze and include their anonymous clinical medical records in clinical research.

## Author Contributions

RH took the lead in designing, writing and revising the manuscript with input from RT. RH double-checked collected data, performed statistical calculations, analyzed the data, and critically interpreted the results. P-HT supervised the project. All authors discussed the results, read and approved the final manuscript.

## Conflict of Interest

The authors declare that the research was conducted in the absence of any commercial or financial relationships that could be construed as a potential conflict of interest.
